# Effects of prenatal inhalation exposure to copper nanoparticles on murine dams and offspring

**DOI:** 10.1186/s12989-015-0105-5

**Published:** 2015-10-06

**Authors:** Andrea Adamcakova-Dodd, Martha M. Monick, Linda S. Powers, Katherine N. Gibson-Corley, Peter S. Thorne

**Affiliations:** Department of Occupational and Environmental Health, University of Iowa, College of Public Health, UI Research Park, IREH 170, Iowa City, IA 52242 USA; Department of Internal Medicine, University of Iowa, Iowa City, IA 52242 USA; Department of Pathology, University of Iowa, Iowa City, IA 52242 USA

**Keywords:** Copper nanoparticles, Prenatal, Inhalation, Toxicity, Pregnancy, Mice, Th1/Th2 profiles, Immunotoxicity

## Abstract

**Background:**

Increasing numbers of individuals may be exposed to nanomaterials during pregnancy. The overarching goal of this investigation was to determine if prenatal inhalation exposure to copper nanoparticles (Cu NPs) has an effect on dams and offspring, including an analysis of inflammatory markers (Th1/Th2 cytokine profiles).

**Methods:**

Physicochemical characterization of Cu NPs was performed. Pregnant and non-pregnant mice (C57Bl/6 J) were exposed to Cu NPs or laboratory air in the whole-body chamber for 4 hrs/day on gestation days (GD) 3–19 (3.5 mg/m^3^). Animals were euthanized on GD 19 (0 week) or 7 weeks later. Bronchoalveolar lavage (BAL) fluid was analyzed for total and differential cells. Cytokine/chemokine concentrations were determined in the BAL fluid and the plasma of dams/non-pregnant mice and pups. Cu content was determined in the lungs and the blood of dams/non-pregnant mice and pups, in the placentas as well as in the whole bodies of pups immediately after delivery. Lungs and placentas were evaluated for histopathological changes. Gene expression of the Th1/Th2 profiles were analyzed in spleens of pups.

**Results:**

The survival rate of 7 week old pups exposed to Cu NPs was significantly lower than control pups (73 vs. 97 %). The average litter size, male/female ratio, body weight and lenght at birth were not different between Cu NP-exposed and control mice. Both pregnant and non-pregnant mice exposed to Cu NPs had significant pulmonary inflammation with increased number of neutrophils in the BAL fluid compared to controls. Perivascular lymphoplasmacytic cuffing was found in the lungs of exposed mice and was more pronounced in the non-pregnant group. Similarly, levels of inflammatory cytokines/chemokines IL-12(p40), G-CSF, GM-CSF, KC, MCP-1, MIP-1α, MIP-1β, RANTES and TNF-α in BAL fluid were significantly higher in non-pregnant than pregnant exposed mice. Histopathology evaluation of placentas did not identify any pathological changes. No translocation of Cu into the placenta or the fetus was found by inductively coupled plasma-mass spectroscopy. Expression of several Th1/Th2 or other immune response genes in pups’ spleens were found to be significantly up- or down-regulated.

**Conclusions:**

Prenatal exposure to Cu NPs caused a profound pulmonary inflammation in dams and strong immunomodulatory effects in offspring. There was no clear polarization of genes expressed in pups’ spleens towards Th1 or Th2 type of response.

**Electronic supplementary material:**

The online version of this article (doi:10.1186/s12989-015-0105-5) contains supplementary material, which is available to authorized users.

## Background

Human exposure to ultrafine particles in ambient air has been associated with adverse health outcomes including pulmonary and cardiovascular diseases [1–3], lung cancer [4], allergy and with adverse pregnancy outcomes such as premature birth, reduced birth weight, small size for gestational age and still birth [5]. Animal studies demonstrate that *in utero* exposure to fine and ultra-fine particles leads to multiple deleterious immunological changes in offspring, such an inhibition of Th1 maturation and postnatal asthma development [6, 7]. It has been shown that in many cases nanoparticles (NPs) are taken up by cells and induce production of pro-inflammatory cytokines and can likely have immunomodulatory effects on the exposed organism [8].

With the fast development of nanotechnology the potential risks of NP exposure to human health is expanding. A wide variety of consumer products already contain a varying array of nanomaterials including health and fitness products, targeting pharmaceuticals, cosmetics, antibacterial clothes, home and garden products, electronics and computers, and paints. Therefore, chances for exposure to engineered nanomaterials are increasing for the public as well as for individuals in the occupational environment. Inhalation is one potential route of exposure and can occur during manufacturing, accidental release of materials, use of consumer products or medical applications. Even though it is a small percentage of the population, the most vulnerable and sensitive group to the adverse effects of NP exposure are pregnant women and their developing fetuses. However, there is limited information about the effects of exposure to nanomaterials during pregnancy. The risk of these materials is not only related to the manufactured quantities and the probability of exposure but also on the reactivity and potency to biological systems, including the immune system [9]. Increased inflammation during pregnancy (including production of a number of pro-inflammatory cytokines) may negatively influence the normal fetal development and may have negative postnatal consequences.

Copper and copper oxide nanoparticles have been used and investigated for a large number of applications, such as oxidation catalysts and as a component of solar cells [10], as well as being the major element of conductive inks and pastes used in inkjet-printed electronics [11]. Other applications are being developed due to their anti-microbial, anti-biotic and anti-fungal properties when incorporated in coatings, plastics and textiles and for biomedical applications [12, 13].

In the last several years, an increasing number of studies have investigated the possibility of transport of nanomaterials *via* placenta [14–16] and their effect on the fetus [17–22]. If NPs cross the placenta, they might result in a direct effect on the fetus. Indeed an *ex vivo* study of barrier capacity of human placenta found that polystyrene particles (50, 80, 240, and 500 nm) were able to penetrate the placental barrier [15]. Similarly, an *in vivo* study in mice showed that quantum dots crossed the placental barrier [17]. There might also be indirect effects of prenatal inhalation exposure to NPs on the developing fetus. Up to now, there are a limited numbers of studies that focused their research on *in utero* effects of NPs after repeated inhalation exposure [18, 19, 23]. These studies show that even after inhalation exposure during gestation, NPs can directly or indirectly affect offspring. Inhaled cadmium oxide NPs negatively impacted fetal and neonatal development and growth [18]. Inhalation of nano-sized coated TiO_2_ during gestation caused lung inflammation in adult mice and neurobehavioral changes in offspring [19]. *In utero* pulmonary exposure to carbon black induced liver DNA damage in dams and offspring [23]. Furthermore, as it was addressed recently by Hougaard et al. [24], there is a lot of uncertainties about embryo-fetal development and health later in life after inhalation exposure to NPs and more studies in this area are warranted.

Normal pregnancy is a complex immunological state. There is a shift from Th1 to Th2 responses that functionally induces maternal tolerance and immunosuppression and protects the fetus [25]. Since nanomaterials have varying biological properties, each NP can cause disparate modulation of the immune system. For example, two catalytically different metallic NPs have shown nearly opposite effects on Th1/Th2 polarization. While titanium dioxide (TiO_2_) NPs potentiated polarization towards Th1-responses, cerium oxide (CeO_2_) induced Th2-dominated responses [8].

The objective of this study was to assess the effects of inhalation exposure to Cu NPs on dams and offspring *in utero* using a murine pregnancy model. Based on previous studies investigating Cu NPs [26, 27], we expected robust inflammatory responses in particle-exposed mothers. We sought to determine if prenatal exposure to inhaled Cu NPs had an effect on 1) basic gestational and developmental parameters, 2) immune/inflammatory responses in the mother during pregnancy compared to non-pregnant mice, 3) translocation of Cu NPs through placental barrier into the fetus, 4) basic pathology of placenta, and 5) immune responses in offspring (expression of Th1/Th2 and other immune response genes in spleens).

## Results

**Fig. 1:**
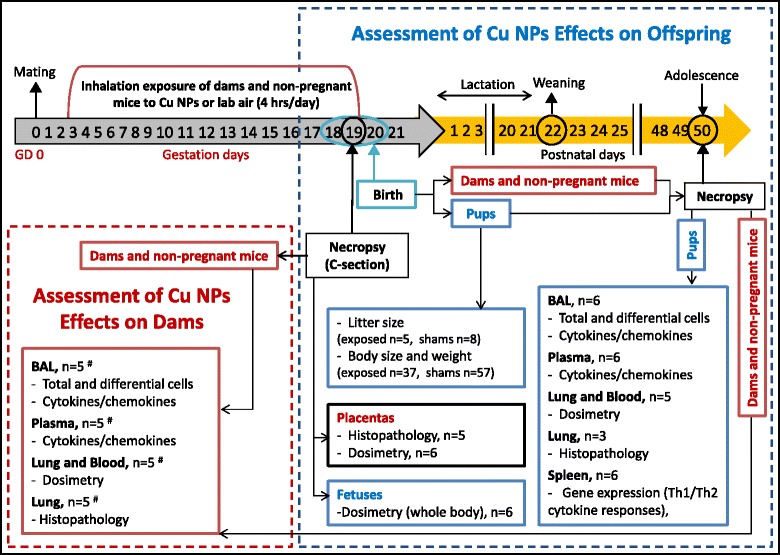
The experimental study design and the number of animals per each evaluated endpoint. All mice (dams and non-pregnant mice) were exposed to Cu NPs or filtered laboratory air (4 hrs/day) from gestation day (GD) 3 to 19. A selected number of mice was euthanized on GD 19 and the pups were delivered by Caesarian section (0 wk post exposure). Another group of mice delivered the pups spontaneously on GD 18–20, these pups grew until adolescence when they were euthanized at 50 postnatal days (PND). ^#^ There were 4 mice in the pregnant group exposed to Cu NPs, all other experimental groups had 5 animals

The experimental design, exposure timeline as well as number of animals per each time point shown in Fig. 1 highlights evaluation of dams, non-pregnant mice and pups immediately after prenatal exposure to Cu NPs and 50 days later when adolescent pups were evaluated. There were 4 experimental groups: pregnant exposed, pregnant control, non-pregnant exposed and non-pregnant control group. Non-pregnant mice received the same exposure and were necropsied at the same time as pregnant groups.

### Physicochemical characterization of NPs

**Fig. 2:**
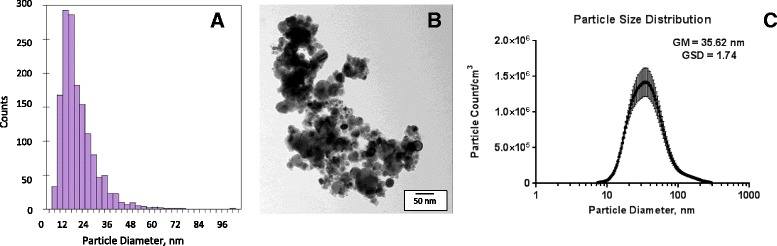
Characterization of Cu NPs. Particle size distribution of primary Cu NPs (**a**) TEM image of primary Cu NPs (**b**), and the size distribution of generated Cu NP aerosol in the whole-body exposure chamber (**c**)

The size of the Cu NPs evaluated by TEM (Fig. 2a, b) showed an average primary particle size of 15.7 ± 9.6 nm (smaller than the manufacturer’s stated size of 25 nm). The surface area of primary particles measured by BET analyses was 14.6 ± 0.5 m^2^/g (Table 1). Powder XRD of the nanomaterial characterized immediately after opening the sealed package containing the nanoparticles showed the presence of metallic Cu along with the presence of Cu_2_O (cuprite). During handling the nanomaterial for aerosol generation and during storage, Cu NPs powder was exposed to the ambient environment. XRD analyses of these particles showed they aged and further oxidized leading to the presence of two phases: the metallic copper phase and a more oxidized coating of CuO (tenorite) on the surface of the particle. The average mobility diameter of airborne particles in the exposure chamber was 35.6 nm with GSD = 1.7 nm (Table 1, Fig. 2c).

**Table 1:** Physicochemical characterization of Cu NPs

Particle characteristic	Cu NPs
Primary particle diameter	15.7 ± 9.6 nm
BET surface area	14.6 ± 0.5 m^2^/g
Particle size in the exposure chamber	GM = 35.6 nm
	GSD = 1.7 nm
Exposure aerosol concentration	3.5 ± 1.2 mg/m^3^

### Gestational parameters

**Fig. 3:**
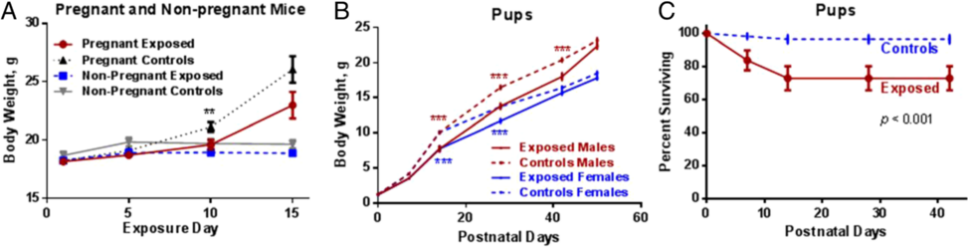
Body weights of all animals and survival rate of pups. The body weights of pregnant and non-pregnant mice during Cu NPs exposure (**a**). Postnatal body weights of pups (**b**) and postnatal survival rate of pups after prenatal inhalation exposure to Cu NPs (**c**). Significant value **p < 0.1 in **a** represents the comparison between pregnant exposed vs. pregnant controls. Significant value ***p < 0.001 in **b** represents the comparison between exposed males vs. control males and exposed females vs. control females pups

During the time of exposure, pregnant exposed mice gained significantly less weight than pregnant controls (*p* < 0.05, ANOVA for repeated measures). In non-pregnant groups, there was no statistical difference in weight gain (Fig. 3a). Both male and female pups with prenatal exposure to Cu NPs gained significantly less weight during postnatal days than their control counterparts (Fig. 3b). Survival rate of pups was significantly (*p* < 0.001) lower in exposed mice than controls (Fig. 3c, Table 2). Only 73 % of pups with prenatal exposure to Cu NPs survived up to 50 PNDs compared to 97 % of controls. All of the pups that died were lost in the first 2 weeks (wks) after delivery, with 16 % loss at 1 week (wk) and 27 % loss at 2 wks after delivery. The length of gestation as well as average litter size was slightly lower in exposed mice than controls, but not significantly. There were no other significant differences between exposed and control mice in the following parameters (Table 2): gestation length, average litter size, male/female ratio, birth weight, and birth body length. Each dam delivered between 6–9 pups with an average litter size of 6.2 ± 1.1 in exposed group and 7.1 ± 2.7 in control group (means ± standard deviations).

**Table 2:** Gestational parameters

Spontaneous birth:	Gestation length, days ± SD	Average litter size, ± SD	Male/female (ratio)	Birth weight ± SE, g	Body length at birth ± SE, mm	Survival rate at 50 PNDs
Exposure	18.4 ± 0.6	6.2 ± 1.1	14/13 (1.08)	1.22 ± 0.01	25.00 ± 0.16	73 %***
Controls	19.0 ± 0.0	7.1 ± 2.7	29/26 (1.12)	1.28 ± 0.01	24.93 ± 0.13	97 %

*SD* standard deviation

PND - postnatal days

***p < 0.001 significantly lower survival rate compared to controls

### Evaluation of BAL fluid cells

#### Pregnant and non-pregnant mice

**Fig. 4:**
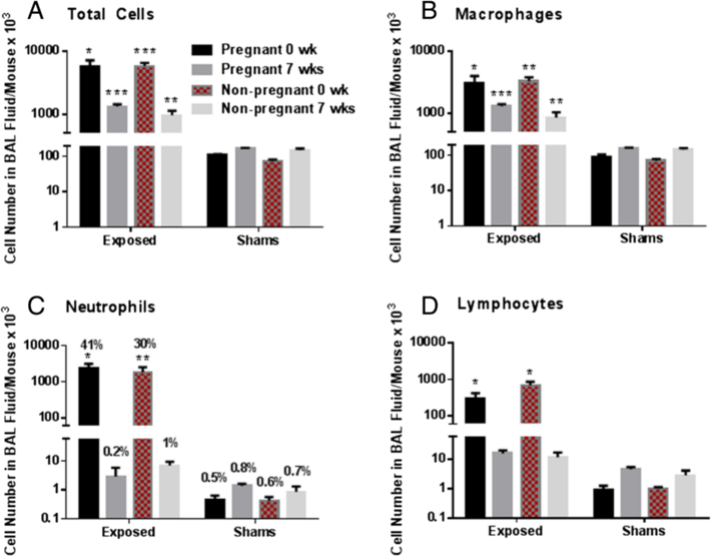
Cell numbers in BAL fluid of exposed and control pregnant and non-pregnant mice. A comparison of the number of total cells (**a**), macrophages (**b**), neutrophils (**c**) and lymphocytes (**d**) in the BAL fluid in pregnant and non-pregnant mice exposed to Cu NPs and their sham counterparts. **c** the percentage of neutrophils out of the total number of cells is also shown. Values are expressed as mean ± SE. Statistically significant differences between exposed groups and control counterparts (shams) are indicated as follows: *p < 0.05, **p < 0.01 and ***p < 0.001

The total number of white blood cells recovered by BAL at 0 wk after exposure was significantly higher in Cu-NP-exposed pregnant (5710 ± 1550 × 10^3^ cells per mouse) or non-pregnant mice (5840 ± 720 × 10^3^ cells per mouse) compared to their sham counterparts (*p* < 0.05 and *p* < 0.001, respectively, Fig. 4a). This major increase was 53-fold in pregnant and 81-fold in non-pregnant Cu-NP-exposed mice compared to their controls. The number of cells decreased 7 wks after the exposure, but there was still a significant 8-fold (*p* < 0.001) and 6-fold (*p* < 0.01) increase in exposed pregnant and non-pregnant mice, respectively compared to their controls. There was no significant change in recruitment of total cells into the lungs between exposed pregnant and non-pregnant mice. The number of macrophages in BAL fluid showed a similar trend as the total number of cells, with the highest recruitment in non-pregnant Cu-NP-exposed mice at 0 wk post exposure (3314 ± 538 × 10^3^ cells per mouse), followed by pregnant Cu-NP-exposed group (3083 ± 937 × 10^3^ cells per mouse) at 0 wk post exposure. The number of macrophages was significantly higher in both exposed groups compared to shams (Fig. 4b).

Recruitment of neutrophils into the airways immediately after the last exposure was also significantly increased in both pregnant (2320 ± 820 × 10^3^ cells per mouse, *p* < 0.05) and non-pregnant (1850 ± 670 × 10^3^ cells per mouse, *p* < 0.01) exposed groups compared to their pregnant and non-pregnant controls (0.5 ± 0.2 and 0.4 ± 0.2, respectively). At 7 wks post exposure, the number of neutrophils was found to be similar to controls (Fig. 4c). The number of lymphocytes in BAL fluid per mouse at 0 wk after exposure was significantly (*p* < 0.05) increased (Fig. 4d) in exposed pregnant (304 ± 114 × 10^3^) and non-pregnant mice (681 ± 180 × 10^3^) compared to pregnant and non-pregnant controls (0.9 ± 0.4 × 10^3^ and 1 ± 0.1 × 10^3^, respectively). No eosinophils were found in BAL fluid.

#### Pups

**Fig. 5:**
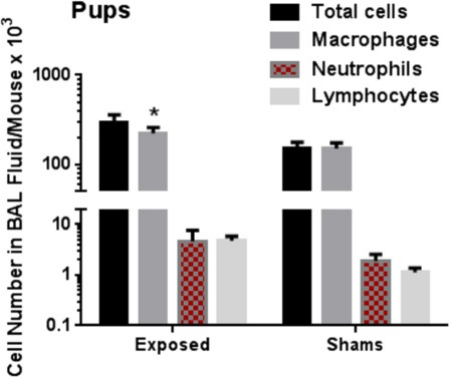
Cell numbers in BAL fluid of exposed and control pups. The total cell number and number of macrophages, neutrophils and lymphocytes in the BAL fluid of mice at 50 PNDs that were exposed to Cu NPs during gestation days 3–19. Values are expressed as mean ± SE. Statistically significant difference between the exposed group and the control group is indicated as *p < 0.05

Spontaneously delivered pups were necropsied at 50 PND. The total number of cells in BAL fluid was increased in pups with prenatal exposure to Cu NPs (293 ± 66 × 10^3^ cells per mouse) compared to controls (155 ± 23 × 10^3^ cells per mouse), but this increase was not significant (*p* < 0.06). The number of macrophages was significantly (*p* < 0.02) increased in prenatally exposed pups (223 ± 36 × 10^3^ cells per mouse), compared to controls (152 ± 22 × 10^3^ cells per mouse) while the number of neutrophils and lymphocytes in BAL fluid did not differ from controls (Fig. 5). No eosinophils were found in BAL fluid.

#### Cytokines in BAL fluid and plasma

**Fig. 6:**
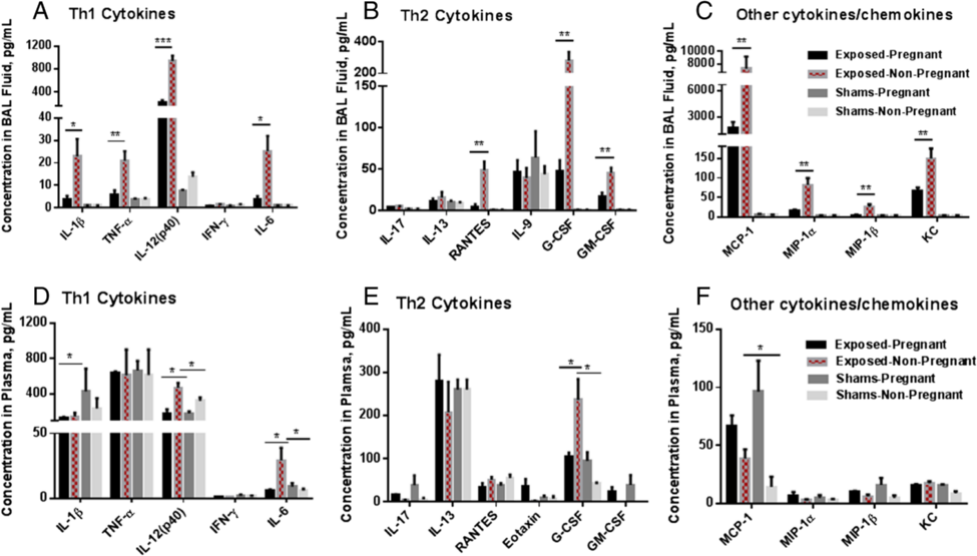
Cytokine/chemokine concentrations. The concentration of cytokines/chemokines in the BAL fluid (**a**-**c**) and the plasma (**d**-**f**) of pregnant and non-pregnant mice exposed to Cu NPs during gestation and their control counterparts divided into functional groups as follows: Th1 cytokines (**a** and **d**), Th2 cytokines (**b** and **e**) and other cytokines/chemokines (**c** and **f**). Values are expressed as mean ± SE. Statistically significant differences between the exposed groups and the control counterparts (shams) are indicated as follows: *p < 0.05, **p < 0.01 and ***p < 0.001

An array-based analysis panel of 23 cytokines/chemokines was used to analyze supernatants of BAL fluid from dams/non-pregnant mice at 0 day post exposure and pups at 50 PNDs. Of the proteins tested from BALs, some cytokines were below lowest limit of detection: IL-1α, IL-2, IL-3, IL-4, IL-5 and IL-12(p70). The concentration of IL-10 was very low in non-pregnant groups and not detected in pregnant mice. Cytokines that were detectable in adult females’ BAL fluid were higher in both exposed groups of mice (pregnant and non-pregnant) compared to controls. The concentrations of the following cytokines were significantly higher in exposed non-pregnant mice compared to exposed pregnant mice: IL-1β, TNF-α, and IL-6 (in Th1 group, Fig. 6a), RANTES, G-CSF and GM-CSF (in Th2 group, Fig. 6b), MCP-1, MIP-1α, MIP-1β and KC (in other cytokines/chemokine group, Fig. 6c). The highest chemokine level in BAL fluid among Cu-NP-exposed mice was MCP-1, with a 4-fold higher concentration in non-pregnant mice (7930 ± 1880 pg/mL) compared to pregnant mice (1710 ± 700 pg/mL). The second highest concentration was IL-12(p40) with a 4-fold higher concentration in exposed non-pregnant mice (940 ± 100 pg/mL ) compared to exposed pregnant mice (220 ± 40 pg/mL). For the pups, the majority of the cytokines/chemokines were not detected with the exception of IL-9, IL-12(p40), IL-13, IL-17, IFN-γ, KC, and TNF-α. However there were no significant concentration differences in pups with prenatal Cu-NP-exposure *vs.* controls (data not shown).

The same array of cytokines/chemokines was analyzed in the plasma of dams/non-pregnant mice and pups. Cytokines that were significantly elevated in the plasma of non-pregnant exposed mice compared to non-pregnant controls as well as compared to pregnant mice exposed to Cu NPs included: IL-12(p40), IL-6, and G-CSF (Fig. 6d and e). The concentrations of cytokines/chemokines in the plasma of pups were very low and there were no significant differences between exposed and control pups (data not shown).

### Histopathology of lungs and placenta

**Fig. 7:**
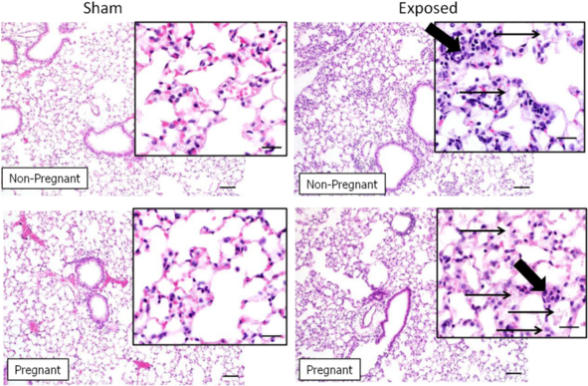
Lung histopathology. Lung from mice exposed to Cu NPs and euthanized immediately after C-section on GD 19 (0 PND) exhibited histopathologic changes including mild to moderate, multifocal inflammatory cell infiltration within the interstitium which is composed primarily of lymphocytes and neutrophils with fewer plasma cells and macrophages (*thick arrows*). Also present was multifocal accumulations of a basophilic, flocculent, amorphous material (*thin arrows*). The pathology findings were more pronounced in non-pregnant mice as opposed to the pregnant group. There were no pathologic findings in control mice. Bars = 200 μm (inset, bars = 20 μm)

Lung tissue evaluations of controls and exposed dams/non-pregnant mice showed a negative effect of NP inhalation in all exposed groups compared to controls. The pathology findings were minor but more pronounced in non-pregnant mice as opposed to the pregnant group (Fig. 7 and Table 3). There were varying amounts of amorphous alveolar debris and foamy alveolar macrophages in the lungs of exposed mice, both pregnant and non-pregnant. Neutrophilic alveolar inflammation, represented by multifocal pockets of neutrophils within the lungs, was only found in non-pregnant exposed mice at day 0. Similarly, perivascular lymphoplasmacytic cuffing was most distinct in the non-pregnant day 0 group, but was also present in both exposed groups at day 50 post exposure. Cuffing at day 50 was less prominent than at 0 day post exposure and was more multifocal. There were no notable pathology findings in either the lungs from the pups or the placentas in either the sham or the exposed groups. The morphology of major vessels and capillaries in placenta were evaluated as well as the labyrinth, decidual and spongiotrophoblast layers were measured in all placental tissues and no significant differences were noted (Additional file 1).

**Table 3:** Histopathology scores of exposed and control pregnant and non-pregnant groups immediately after delivery on GD 19 (PND 0) and 50 days later (PND 50)

Experimental Group		Number of mice per group	Histopathology Score^a^		
			Alveolar macrophages	Alveolar debris	Perivascular cuffing^b^
Exposed	Pregnant, PND 0	*n* = 4	1	2	0.75
	Non-Pregnant, PND 0	*n* = 5	1	1	1.8
	Pregnant, PND 50	*n* = 5	1	0	0.2
	Non-Pregnant, PND 50	*n* = 5	0.6	0	0.2
Shams	Pregnant, PND 0	*n* = 4^c^	0	0	0
	Non-Pregnant, PND 0	*n* = 4^c^	0	0	0

*PND* postnatal day

^a^Scoring system: 0 = none, 1 = few/rare, 2 = multifocal/moderate, 3 = many/coalescing and/or diffuse

^b^Lymphocytes and plasma cells surrounding blood vessels

^c^Only 4 mice out of 5 were evaluated for pathology changes in control groups

### Determination of Cu levels

Cu was measured by ICP-MS in the whole blood and in the lungs of pregnant and non-pregnant mice, placentas, fetuses delivered by Caesarian section (C-section) at GD 19, and pups (whole blood) at 50 PND (Table 4). There was no significant difference in Cu concentration in the whole blood between exposed dams and controls. Both groups of pregnant mice, exposed as well as shams, had a 2-fold increase in Cu levels in the whole blood, compared to non-pregnant mice. Interestingly, the inhalation of Cu NPs did not elevate Cu concentrations in the blood of exposed animals. The levels of Cu in the lung tissue of exposed pregnant mice (85.9 ± 3.9 μg/L) at 0 PND were increased significantly (*p* < 0.001) compared to pregnant shams (23.4 ± 4.1). Also highly significant elevation (*p* < 0.0001) of Cu levels in the lungs was found in non-pregnant exposed (54.4 ± 2.8 μg/L) mice compared to non-pregnant shams (13.0 ± 1.2 μg/L). The levels of Cu in lung tissue of exposed pregnant and non-pregnant mice were 3.7- and 4.2-times higher, respectively, compared to controls. Cu concentrations in the blood as well as in lungs were found to be lower at 50 PND compared to 0 PND, but there was still 2.2- and 1.7- times increase in exposed pregnant and non-pregnant mice, respectively, compared to controls.

**Table 4:** Dosimetry of Cu NPs in the whole blood, lung and placenta

Experimental group		Cu Concentration, Mean ± SE					
		Whole blood, μg/L		Lungs, mg/kg		Placenta, mg/kg	Fetus (GD19), mg/kg
Adult females		PND 0	PND 50	PND 0	PND 50		
Exposed	Pregnant	1400 ± 91.7^###^	644 ± 7.0	85.9 ± 3.9***, ^###^	28.5 ± 3.4**, ^##^	19.9 ± 1.8	11.6 ± 0.3
	Non-Pregnant	737 ± 26.4	669 ± 20.7	54.4 ± 2.8***	20.0 ± 0.6***	--	--
Shams	Pregnant	1450 ± 18.3	ND	23.4 ± 4.1	12.8 ± 1.0	17.6 ± 1.6	11.1 ± 0.5
	Non-Pregnant	704 ± 13.2	ND	13.0 ± 1.2	11.8 ± 0.4	--	--
Pups							
Exposed		--	600 ± 18.9	ND	ND		
Shams		--	655 ± 30.0	ND	ND		

*ND* not determined

^###^ *p* < 0.001, ^##^ *p* < 0.01 significant difference between pregnant and non-pregnant mice

***p < 0.001, **p < 0.01 significant difference between exposed and control counterparts

There was no significant difference in Cu levels in placentas (19.9 ± 1.8 mg/kg *vs.* 17.6 ± 1.6 mg/kg), fetuses (whole body) at GD 19 (11.6 ± 0.3 mg/kg *vs*. 11.1 ± 0.5 mg/kg) or in the whole blood of pups at 50 PND (600 ± 18.9, μg/L *vs.* 655 ± 30.0, μg/L) between exposed group and controls, respectively.

### Gene expression analyses

**Fig. 8:**
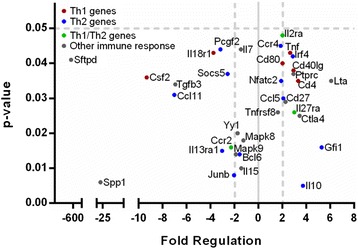
Spleenic gene expression in pups. The mouse Th1 and Th2 responses were identified in RNAs isolated from the spleens of pups after prenatal exposure to Cu NPs by RT^2^ Profiler PCR Array (PAMM-034ZA, SABiosciences, Qiagen). Out of 84 genes, 14 genes were significantly (*p* < 0.05) up-regulated at least 2-fold and 11 genes were significantly (*p* < 0.05) down-regulated at least 2-fold from the controls

The mRNA expression of 84 genes involved in T-helper cell (Th cell) immune responses was determined in spleenic mRNAs of pups at 50 PNDs with prenatal exposure to Cu NPs and compared to control pup spleenic mRNA. Out of 84 genes, 14 genes were significantly (*p* < 0.05) up-regulated at least 2-fold from controls and 11 genes were significantly (*p* < 0.05) down-regulated at least 2-fold from controls (Fig. 8). The two highest up-regulated genes were *Lta* (Lymphotoxin A, 6-fold) and *Gfi1* (Growth factor independent 1, 5-fold). The most significantly down-regulated genes were *Sftpd* (Surfactant associated protein D, 630-fold, *Spp1* (Secreted phosphoprotein 1, 27-fold), *Csf2* (Colony stimulating factor 2 [granulocyte-macrophage], GM-CSF, 9-fold), *Ccl11* (Chemokine (C-C motif) ligand 11, eotaxin, eosinophil chemokine, 7-fold), and *Tgfb3* (Transforming growth factor, β 3, 7-fold). There was no clear polarization towards Th1- or Th2-related genes. Table 5 lists only genes with fold-change > = 2 or p-value < 0.1.

**Table 5:**
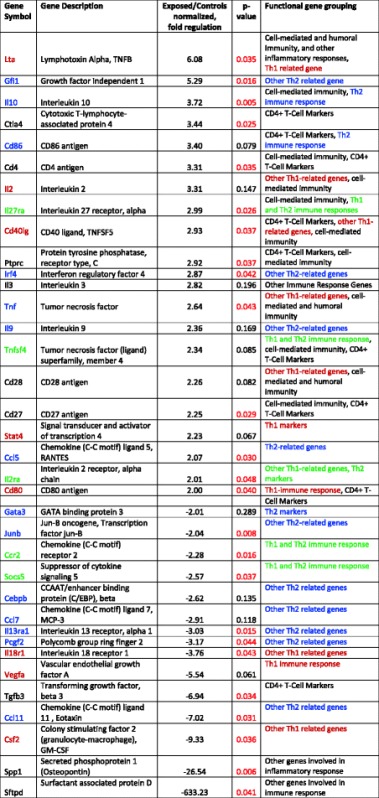
Gene expression changes in Cu NP-exposed and control pups, spleen tissue (rtPCR array)

Out of all 84 genes, only genes with fold-change > = 2 are listed in this table. Genes are color-coded according to a response, red: Th1 related genes, blue: Th2 related genes, green: Th1 and Th2 responses, black: other genes involved in immune response

## Discussion

Regardless of the numerous benefits of nanotechnology applications, there is a potential health threat from nanoparticle exposure that needs to be addressed also in more susceptible populations, including pregnant women and developing fetuses. Exposure to NPs during sensitive developmental stages of life may predispose an organism to diseases later in life. In our study, we assessed the effects of prenatal inhalation of Cu NPs on dams and offspring using a mouse model.

We performed physicochemical characterization of commercially available Cu NPs immediately after opening the package prior to exposure to the ambient environment, as well as after exposure that occurred during storage and handling. The primary particle size evaluated by TEM was 15.7 ± 9.6 nm. As expected and observed previously, Cu NPs can readily oxidize upon exposure to the ambient environment and this can change the properties of the original nanoparticles. The dynamic nature of Cu NPs is described in Mudunkotuwa *et al*. [28]. Oxidation of Cu NPs was also observed in our study during storage and handling. Initial characterization of Cu NPs minimally exposed to the ambient air showed the presence of mainly metallic Cu with a thin coating of Cu_2_O (cuprite) on the particle surface as shown by XPS. The XRD pattern of material exposed to the ambient environment showed the presence of two phases: metallic Cu and CuO (tenorite) only with no Cu_2_O phase. An average mobility diameter of airborne particles in the whole-body chamber was 35.6 nm with GSD = 1.7 nm.

At present, there is not enough information available to determine a potential hazard of prenatal exposure to NPs especially by inhalation. Furthermore, the exposure limits are nowadays available only for a limited number of nanomaterials (such as TiO_2_ and carbon nanotubes). Since there are currently no exposure limits for Cu NPs, we based our animal exposure studies on the current exposure limits for coarse copper material. The Recommended Exposure Limit (REL) for copper dust and mist (not including fumes) established by the National Institute for Occupational Safety and Health (NIOSH) as well as Permissible Exposure Limit (PEL) by the Occupational Safety and Health Administration (OSHA) is 1 mg/m^3^ as a time-weighted average concentration. The estimated dose of Cu NPs in the tracheobronchial and pulmonary region after exposure during gestation was 141 μg/mouse (6.1 mg/kg). This estimated dose corresponded to the lung dose accumulated by a human (70 kg) exposed to a concentration of 1 mg/m^3^ for a period of 39.7 work wks (assuming breathing frequency of 15 breaths/min, 600 mL/breath, and pulmonary deposition fraction for 36 nm particles of 0.5) [29]. The average length of a human pregnancy is 40 wks.

### Basic gestational and developmental parameters were affected by prenatal Cu NP exposure

Prenatal inhalation exposure to Cu NPs during GD 3 – 19 had a significant effect on some basic developmental and gestational parameters. Maternal weight gain during gestation was lower in exposed pregnant mice than in controls. Similarly, pups with prenatal exposure gained weight slower than the control pups. Length of gestation, as well as litter size were also slightly (not significantly) affected by the exposure; all other basic gestational measures were not affected. It is possible that with the increased sample size per each group, these observations could become significant. The survival rate of pups at 50 PNDs was significantly lower in exposed mice than controls. Another study investigating prenatal inhalation exposure of TiO_2_ particles (rutile) on GD 8–18, 1 hr/day, concentration of 42 mg/m^3^, found no effects on maternal weight gain, length of gestation, loss of implantations, litter size, offspring body weight or sex ratio. Pup survival during the lactation period was not significantly reduced in TiO_2_ litters [19]. Intra-tracheal exposure to carbon nanoparticles on GDs 7 – 14 (200 μg/mouse per each day) did not affect basic gestational parameters [30]. This suggests that Cu NPs have greater toxicity for fetuses than some other nanomaterials. The observed lower survival rate of pups with prenatal exposure to Cu NPs could potentially be due to reduced food intake by exposed dams caused by inflammation, secondary exposure to Cu NPs or Cu ions via lactation, or disruption/alteration of immune, inflammatory, metabolic, biochemical or endocrine pathways in offspring. However, our study only explored inflammation and immune pathways and does not offer an explanation for this adverse effect.

### Cu NP exposure during gestation affected immune/inflammatory responses in mothers

We found significantly higher inflammatory responses in the lungs of both pregnant and non-pregnant mice exposed to Cu NPs during GD 3 – 19 compared to pregnant and non-pregnant shams. This was represented by significantly higher numbers of total leukocytes and neutrophils in the BAL fluid. Production of Th1, Th2 or other cytokines/chemokines in BAL fluid was significantly higher in the non-pregnant group exposed to Cu NPs than in the exposed pregnant group. Exposed non-pregnant mice had also a higher systemic inflammation than pregnant exposed mice. Our data suggest that the Th2 pregnancy environment partially protected the lungs of the pregnant mice from the pro-Th1 effects of the Cu NPs. The histopathology score of perivascular cuffing (represented by lymphocytes and plasma cells surrounding blood vessels) in nanoparticle-exposed mice was also 2.4-fold higher in non-pregnant (1.8) than pregnant mice (0.75). We did not observe a clear polarization of Th1 or Th2 cytokines in BAL fluid of dams exposed to Cu NPs during gestation.

Several animal studies have reported on differences in pulmonary inflammatory responses among pregnant and non-pregnant rodents. Contrary to our study presented here, several previous studies found pregnant rodents to be more susceptible to various inflammatory stimuli than non-pregnant animals. For example, pregnant rats had an enhanced inflammatory response to inhaled endotoxin [31] or acute ozone exposure [32] when compared to non-pregnant controls. Similarly, much stronger pulmonary inflammation was observed in pregnant mice than in non-pregnant females after intra-nasal instillation of TiO_2_ or diesel exhaust particles [7]. The enhanced inflammation in pregnancy after TiO_2_ particle exposure was recently associated with impaired uptake of particles by macrophages due to estradiol release during pregnancy [33].

Based on our previous results [26, 27], we expected that Cu NP inhalation exposure would cause an immense inflammatory response; however, we wondered whether the pregnancy phenotype (characterized by predominant Th2 response) would have an effect on this inflammatory response in pregnant vs. non-pregnant mice. Furthermore, we sought to explore if enhanced inflammation during pregnancy would modulate the immune system of the developing fetus. The immunomodulatory effects of various nanomaterials have been reported previously, e.g. maternal exposure to combustion-generated ultrafine particles inhibited pulmonary Th1 maturation and enhanced asthma development in offspring [6]. Indeed, we observed a significant inflammatory response in the lungs of exposed animals. However, this response was larger in the non-pregnant mice than in their pregnant counterparts. This finding might be explained by the fact that pregnancy dominated with Th2 response is a state of maternal suppression with anti-inflammatory effects, and thus, the competition between anti-inflammatory effects of pregnancy and inflammatory effects of Cu NPs may have occurred. Suppression of allergic pulmonary inflammation was also found in asthmatic mice that were exposed to fine or nanosized TiO_2_ particles suggesting that the immunological status of the exposed mice determines the modulation of the airway inflammation [34]. To offer more definite explanations for these findings, studies with different types of nanomaterials with various physicochemical properties and further evaluation of inflammation and endocrine pathways are warranted. Recently, a study by Jones et al. [35] suggested that Th1-Th2 balance is an important factor in NP clearance, the authors demonstrated an increased clearance of NPs in Th2-prone mice. However, clearance of particles is also dependent on the physicochemical properties of nanomaterials as mentioned earlier. Furthermore, in our study there was a physiological increase of Cu levels during pregnancy and thus, assessment of the clearance of particles in our study is more complex. We could not determine how much of the increased Cu levels was due to a physiological increase during pregnancy and how much was due to exposure to Cu NPs.

### No translocation of Cu ions into the placenta or fetus was observed by ICP-MS

Nanoparticle translocation from the lung into circulation and secondary target organs after inhalation exposure has been previously reported [36, 37] and is very much dependent on the particle size, surface area, charge, and functionality, as well as solubility. The translocation of NPs to fetus has been reported as very low and mainly occurring after intravenous exposure [38–40]. Cu NPs were shown to be highly soluble in artificial lung fluid [26]. Thus, we hypothesized that translocation of Cu NPs into the blood stream and possibly to the fetus was more probable than in studies with non-soluble particles. However, our study did not find any increased Cu levels in the placentas or fetuses. Similarly, we did not observe translocation of Cu NP to other tissues in our previous sub-acute inhalation study [27]. The levels of Cu ions in the blood of exposed dams were not significantly higher than in their control counterparts. Pregnancy itself increased the levels of Cu ions in the blood, irrespective of the Cu NP exposure. On the other hand, some of the nanoparticles were likely cleared from the lungs by the mucociliary escalator to the trachea and digestive system or lymphatic system, but this was not determined in this study.

Our result of increased Cu levels in blood during pregnancy is in an agreement with the results of existing human studies [41–44]. Many of them report a 2-fold increase of Cu levels in blood at full term, as was also seen in our study. The increase of Cu levels in lungs of exposed mice was approximately 4-fold, in both pregnant and non-pregnant mice. The levels of Cu in other organs were not determined in this study. It is possible that only a very small percentage of delivered material (in the particle form or in the form of Cu ions after potential dissolution) translocated into the blood and other target organs as was reported in other studies [38, 45, 46] and we were unable to detect the Cu using the ICP-MS method, which was a limitation of this study. Interestingly, we saw retention of Cu in the lungs 7 wks after exposure; there was still 34 and 37 % retained Cu from the original amount found in the lung at the end of gestation in pregnant and non-pregnant exposed groups, respectively.

### No histopathological changes in the placental tissues were found

Inhalation of particles during pregnancy may induce acute placental inflammation [47] that can lead to inadequate placental perfusion and decrease transplacental nutrient exchange [48]. Several studies found that inhaled or injected NPs enter the circulatory system and can migrate to various organs or tissues [49, 50]. Particles containing lead and nickel were found in human placentas from subjects living in proximity to polluted areas [51]. The accumulation of environmental pollutants in the placenta may cause hypoxia that can result in enzymatic changes. The increased activity of lactate dehydrogenase (LDH) was found in human placentas from women residing in areas polluted with fine metal particles [52]. The pollutants captured in the placental tissue may diminish placental function *via* impairment of placental microstructure [53]. Thus, even if the direct translocation of NPs from mother to fetus would not occur, NPs that are taken up by the placenta may cause placental functional changes and subsequently have an impact on the developing fetus. Damaging effects observed in fetuses after i.v. administration of silica NPs were linked to structural and functional changes in the placenta only at the highest concentration of 0.8 mg per mouse [39]. Inhalation of diesel exhaust caused immunological changes in the placentas of exposed mice [54]. Our study did not find any histopathological changes in the placental tissues (Additional file 1). Functional changes of the placentas potentially caused by Cu NP exposure were not evaluated in this study, but since we found a significantly lower survival rate of pups that were prenatally exposed to Cu NPs, as well as changes in immunological responses of exposed mothers, functional changes are not excluded and should be investigated further.

### Prenatal inhalation exposure to Cu NPs induced gene expression changes in Th1/Th2 or other immune responses in spleenic mRNAs in offspring

*In utero* exposure to xenobiotics has been proposed as an important driver for gene reprogramming that may lead to the development of higher vulnerability or resistance to various diseases. The spleen is the largest lymphatic organ and is the primary site of innate and adaptive immune processes, and along with the bone marrow, it is the main organ involved in the manufacturing, maturation, differentiation, proliferation and storage of immune cells.

Of 84 genes involved in T helper cell (Th cell) immune responses that were determined in spleenic RNAs of pups at 50 PNDs with prenatal exposure to Cu NPs, a number stood out as having potential health links. For one, mRNA encoding Surfactant associated protein D (Sftpd) was found to be the most down-regulated (633-fold) compared to controls (*p* < 0.04). Surfactant protein (SP)-D plays an important role in pulmonary innate immunity. But it is also known to be involved in non-pulmonary modulation of inflammation [55]. The presence of SP-D in extrapulmonary tissues has been reported in various mammals [56]. As reviewed by others [57, 58] SP-D mediates many mechanisms such as opsonization of pathogens, activation of phagocytosis and modulation of toll-like receptor (TLR) activity. It was also suggested that surfactant proteins, both SP-A and SP-D, can modulate the immune response to allergens and/or development of allergic reactions [59]. A protective role of SP-D in the pathogenesis of allergic airway disease was found (a study using wild type or SP-D−/− mouse model challenged with house dust mite antigen) [60]. Many other immune roles pulmonary and extrapulmonary are reviewed by Nayak et al. [61].

Another gene that was found to be significantly (*p* < 0.01) down-regulated (27-fold) in prenatally exposed pups was Secreted phosphoprotein 1 (Spp1; also known as Osteopontin). Spp1 was found to modulate immune responses at several levels: recruits cells to sites of inflammation, assists in cell attachment and wound healing, mediates cell activation and cytokine production through interaction with cellular signaling pathways, and it is involved in specific apoptosis pathways. During inflammation it stimulates both pro- and anti-inflammatory processes. Deficiency of this protein is connected to reduced Th1 immune responses in infectious diseases, autoimmunity and delayed type of hypersensitivity [62].

Granulocyte/macrophage colony-stimulating factor (GM-CSF; also known as Csf2) stimulates proliferation, activation, and differentiation of macrophages, granulocytes, neutrophils, eosinophils, and monocytes [63]. The gene of this protein was also found to be significantly (*p* < 0.05) down-regulated (9-fold) in the pups’ spleens.

The three most up-regulated genes in the pups’ spleens were Lymphotoxin-α (Lt-α, also known as tumor necrosis factor-β, [TNF-β]) 6-fold (*p* < 0.05), Gfi1 (Growth factor independent 1) 5-fold (*p* < 0.02), and IL-10 (4-fold, *p* < 0.005).

Lt-α is a cytokine produced by lymphocytes and a member of the TNF family. It is essential in the organogenesis of secondary lymphoid organs (spleen, lymph nodes, and Peyer’s patches) and may induce the development of tertiary lymphoid tissue (accumulation of lymphoid cells in chronic inflammation). Other genes related to the TNF family (ligands or receptors) were also up-regulated, some significantly so, such as Tnf 2.6-fold (*p* < 0.04), Cd40lg (CD40 ligand, TNFSF5) 3-fold (p < 0.03) and Tnfrsf8 (Tnf receptor superfamily, member 8, 1.6-fold, *p* < 0.03) and Tnfsf4 (Tnf (ligand) superfamily, member 4) was up-regulated (2-fold) but not significantly (*p* < 0.09).

Gfi1 (Growth factor independent 1) is a transcriptional repressor that is induced during T-cell differentiation and plays a critical role in enhancing Th2 cell expansion by promoting proliferation and preventing apoptosis. It has been suggested that it works synergistically with Gata3 and provides a mechanism through which IL-4 could promote Th2 cell expansion [64].

The third most up-regulated gene was IL-10, which is known to inhibit inflammatory pathologies and functions at different phases of immune responses and potentially at different anatomical locations. IL-10 was originally recognized as a Th2-type cytokine, but later evidence suggested that it is more relevant to regulatory T (TReg) cell responses and now it is considered a much more broadly expressed cytokine. IL-10 is expressed by many adaptive immune cells including Th1, Th2, and Th17 cell subsets, TReg cells, CD8^+^ T cells and B cells [65]. Due to the fact that various cell types can express IL-10 the regulation of this cytokine is quite challenging. In our study it appears that more pro-inflammatory cytokines were expressed in pups. However, the pathways that induce IL-10 expression may also negatively regulate the expression of these pro-inflammatory cytokines. In many cases, IL-10 is induced together with pro-inflammatory cytokines [65].

## Conclusions

Prenatal exposure to Cu NPs caused a profound pulmonary inflammation in dams with negative effect on pups’ survival after delivery. Many up- and down-regulated genes related to Th1/Th2 type responses point to immunomodulatory effects of Cu NPs in offspring after prenatal inhalation exposure. Our study demonstrated that prenatal exposure to Cu NPs trigger a potent immune Th1/Th2 response in offspring that appear to be activating many pro-inflammatory pathways. However, there was no strong skew either to Th1 or Th2 immune responses in either dams or offspring. We conclude that the changes in expression of immune-response genes in the pups with prenatal Cu NP exposure are due to changes in inflammatory and immune responses of mothers caused by inhalation of Cu NPs rather than due to direct effect of NPs on fetus, since no translocation of Cu was observed through the placenta to the fetus. Further investigation focused on pulmonary prenatal exposure to NPs with postnatal pathogen or allergen challenge, especially in early postnatal stages, might expand our understanding of immunotoxicity potential of metal and metal oxide NPs.

## Materials and methods

### Nanoparticle characterization

Cu NPs with a primary particle size reported by the manufacturer of 25 nm were purchased from Nanostructured and Amorphous Materials, Inc. (Houston, TX, USA) and used without any further intentional modification. The particles were characterized by X-ray diffraction (XRD) using a Bruker D-5000 q – q X-ray diffractometer with a Kevex-sensitive detector (Madison, WI). The primary size of the Cu NPs was assessed by transmission electron microscopy (TEM, JEOL JEM-1230, Japan) by sizing nearly 1500 particles. X-ray photoelectron spectroscopy (XPS) was used to measure the surface chemical composition of the powdered sample (Ultra-Axis XPS, Kratos, Manchester, UK). The surface area was measured with an automated multipoint Brunauer-Emmett-Teller (BET) surface area apparatus using a nitrogen adsorption method (Quantachrome Nova 4200e, Boynton Beach, FL).

### Animals

Mice (C57Bl/6 males and females, 6 wks old) were purchased from Jackson Laboratories (Bar Harbor, ME). All animals were housed in our AAALAC-accredited vivarium, in polypropylene, fiber-covered cages in HEPA-filtered Thoren caging units (Hazelton, PA). They were acclimatized for 10 days after their arrival and were maintained in the vivarium with a 12-hr light/dark cycle with *ad libitum* access to food and water. All protocols performed were approved by our Institutional Animal Care and Use Committee. Animal handling and exposures conformed to the NIH Guide for the Care and Use of Laboratory Animals. Timed pregnancies were established by mating 2 nulliparous females with one single mature male for two days. Each morning the presence of vaginal plug was assessed and if found, the event was considered as gestation day (GD) 1.

### Study experimental design

Pregnant (*n* = 9) and non-pregnant mice (*n* = 10) were exposed to atmospheres of Cu NP-aerosol by inhalation for 4 hr/day in a dynamic whole-body exposure chamber from GD 3 – to GD 19 (total of 17 days). The average mass concentration of nanoparticle-laden aerosol in the whole-body chamber was 3.5 ± 1.2 mg/m^3^ (mean ± standard deviation). Shams, (control mice) pregnant (*n* = 10) and non-pregnant (*n* = 10) were exposed to HEPA-filtered laboratory air in the identical exposure chamber in an adjacent laboratory. The schematic of the study experimental design with the number of mice per each evaluated endpoint is shown in Fig. 1. Pregnant and non-pregnant mice were euthanized with an overdose of isoflurane followed by cervical dislocation, thoracotomy and exsanguination through the heart. One group of dams was euthanized on GD 19 and the pups were delivered by C-section (0 wk post exposure). Another group of mice delivered pups spontaneously on GD 18–20; these pups were euthanized at 50 postnatal days (PND, 7 wks from delivery).

### Generation of NPs and exposure system

The exposure as well as the generation system were described previously [66]. Briefly, a dynamic whole-body exposure chamber was used for animal exposure. Cu NP aerosol was generated from suspension of Cu NPs in water (Optima grade, Fisher Scientific, Pittsburgh, PA) using a 6-jet Collison nebulizer (BGI Inc. Waltham, MA). Suspensions of Cu NPs in water that were placed into the nebulizer were prepared freshly each day by ultra-sonication with a high frequency probe for 20 minutes. A magnetic stir bar was added into the nebulizer to prevent settling the particles. Particle-laden aerosol from the nebulizer passed through a brass drying column heated to 110 °C and particle neutralizer (containing 10 mCi ^85^Kr source, TSI Inc., Shoreview, MN) prior to entering the chamber. Mass exposure concentration of generated aerosol was estimated using a gravimetric method. Pre-weighed fiberglass filters (47 mm in diameter, Whatman, Middlesex, UK) held in a stainless steel sampler were connected to the exhaust outlet from the whole-body chamber and sampled every 2 hrs with a flow of 24 L/min. Filters were pre- and post-weighed in a dedicated climate-controlled room using a microbalance (XP26, Mettler Toledo, Mettler-Toledo, Inc., Columbus, OH).

### Tissue dosimetry

Cu in the blood and lungs from non-pregnant mice and dams immediately after delivery and 50 days later, and in placentas and in pups delivered by C-section on GD 19 were determined using inductively coupled plasma-mass spectrometry (ICP-MS, Perkin-Elmer ELAN DRC II, Norwalk, CT). The method number ITB001A from CDC Laboratory Procedure Manual adapted for digested tissue and Cu was used for these analyses [67]. Whole blood was collected via heart exsanguination into tubes containing EDTA anticoagulant and stored at 4 °C until analyses. Whole blood was then diluted 50 x using a diluent composed of isopropanol (1 % v/v), tetramethyl ammonium hydroxide (1 % v/v), Triton X-100 (0.5 % v/v) and ammonium pyrolidine dithiocarbamate (0.1 % w/v) in deionized water. Lung tissue (left lobe) and placentas were excised and stored at −80 °C. All tissues were then dried using a Speed Vac concentrator (Savant SPD111V, Thermo Scientific, Waltham, MA). Intact carcasses of pups were dried in the oven at 100 °C for 5 hrs. Dry tissues were then weighed and digested in a HotBlock™ digestion system (Environmental Express, Mt. Pleasant, SC) at 95-98 °C using 0.25 mL of high purity concentrated nitric acid (Fisher Optima® grade). After cooling to room temperature, the samples were diluted to 5.0 mL using deionized water. Determination of total Cu in the whole blood and digested tissues was performed using an ICP-MS with high-purity argon (99.99 %), a PFA-ST nebulizer (Elemental Scientific, Omaha, NE) and a 0.50 mm i.d. capillary connected to a cyclonic spray chamber. Sample data were acquired by using 20 sweeps/reading, 1 reading/replicate, and a dwell time of 50 ms. Argon nebulizer gas flow rate was optimized daily from 0.5 to 0.9 L/min. Data were acquired in counts per second (cps). The following isotopes were selected: ^63^Cu and ^65^Cu. Analytical calibration standards were prepared from a single-element stock solution containing 1 g/L of Cu stock solution (SPEX, Metuchen, NJ). For the whole blood measurements, the standards were diluted as follows: 1, 5, 10, 20, 50, 100, 500, 1000, and 2000 μg/L in the same diluent as used for the whole blood. For determination of Cu in tissues and whole bodies, following standard concentrations were used: 1, 5, 50, 100, 500, and 1000 μg/L in 2 % nitric acid. The method detection limit for Cu was less than 1 μg/kg. Rhodium was included in the diluents at 10 μg/L as an internal standard.

### Estimated deposited dose

The estimated pulmonary deposited dose in this study was 141 μg/mouse. We assumed a minute volume of 25 mL/min and a deposition fraction of 0.4 in tracheobronchial and pulmonary region combined. Deposition fraction for 36 nm particles was estimated based on the computational modeling of regional deposition fraction for mice reported by Asgharian et al. [68].

### BAL fluid and plasma evaluation

To assess pulmonary inflammatory response, BAL was performed in dams after C-section and 7 wks later and in 7-wk-old pups. Lungs were washed 3 times with about 1 mL of 0.9 % sterile sodium chloride solution (Baxter, Deerfield, IL). BAL was centrifuged at 800 × g for 5 min at 4 °C, supernatants were frozen at −80 °C for later cytokines/chemokines analyses and cells were resuspended in Hank’s balanced salt solution (Life Technologies, Grand Island, NY). The total number of cells was counted using a hemocytometer. The rest of the cell suspension was fixed on microslides with fetal calf serum (Cytospin 4, Thermo Shandon, Thermo Scientific, Waltham, MA), and stained using Protocol® HEMA 3 stain set (Fisher Diagnostics, Pittsburgh, PA). Differential cell counts (number of macrophages, neutrophils, lymphocytes, and eosinophils) were determined by counting 400 cells using an optical microscope (Olympus, Center Valley, PA). Cytokines/chemokines in BAL fluid supernatants and plasma of dams/non-pregnant mice and pups were analyzed using a multiplex magnetic bead assay (Luminex 100, Bio-Rad Laboratories, Inc., Hercules, CA).

### Gene expression by RT-qPCR (reverse transcription real-time quantitative polymerase chain reaction)

#### RNA Isolation

Lungs and spleens (~30 mg of each tissue) from exposed and unexposed 7-wk-old pups were fixed in RNAlater RNA stabilization reagent (SABiosciences, Qiagen, Valencia, CA, USA) and stored at −20 °C until RNA isolation. Total RNA was extracted using the RNeasy Mini Kit (Qiagen) after tissue homogenization in RLT buffer with the addition of β-mercaptoethanol using a hand-held homogenizer. Genomic DNA was removed utilizing RNase-Free DNase Set (Qiagen). The quality and quantity of a selected number of isolated RNAs were assessed using the Experion automated electrophoresis system (Bio-Rad Laboratories, Inc., Hercules, CA) and the Experion RNA StdSens Analyses kit according to manufacturer’s instructions. The quality of the RNA was considered sufficient if the 28S/18S rRNA (ribosomal RNA) ratio was between 1 and 2 and RNA Quality Indicator (RQI) was > 8. All samples were also checked by the NanoDrop spectrophotometer (Thermo Fisher Scientific, Wilmington, DE); the OD_260_/OD_280_ ratio between 1.8–2.0 was considered acceptable.

Isolated RNA (0.5 μg) was converted to cDNA using the RT^2^ First strand kit and mixed with RT^2^ qPCR SYBR Green mastermix (SABiosciences, Qiagen). Samples were loaded onto a 96-well PCR array (Mouse Th1 & Th2 Responses RT^2^ Profiler PCR Array, PAMM-034ZA) according to manufacturer’s instructions and read using a CFX96 Touch Real-Time PCR Detection System (Bio-Rad Laboratories, Inc., Hercules, CA). The thermocycling conditions were 1 cycle of 95 °C for 10 min (HotStart DNA Taq Polymerase activation) followed by 40 cycles of 95 °C for 15 s and 60 °C for 1 min (fluorescence data collection). Melting curves were performed each time from 55 °C to 95 °C with increments of 0.5 °C for 5 s to ensure that only one amplification product was formed.

### Histopathology

Lungs (right lobes) after BAL were perfused via the cannulated trachea and fixed in 10 % zinc formalin (Fisher Scientific, Kalamazoo, MI). Placentas of dams after C-section delivery were harvested and also fixed in 10 % zinc formalin. Tissues were then paraffin-embedded, sectioned (5-μm thickness) and stained with hematoxylin and eosin (H&E). All tissues were evaluated by a board-certified veterinary pathologist for key histopathologic changes including but not limited to inflammatory cell infiltration, fibrosis and cellular injury and necrosis.

### Statistical analyses

All data are expressed as mean ± standard error (SE) unless otherwise noted. The data were analyzed using a T-test or one-way analysis of variance (ANOVA) followed by a Tukey test if treatment group sizes were equal. Dunn’s test was used in cases when treatment group sizes were unequal. If the Normality Test failed, Kruskal-Wallis one-way ANOVA on Ranks was used. If the Equal Variance test failed, data were analyzed using the Mann–Whitney Rank Sum Test (Sigma Plot v.11.0, Systat Software Inc., Point Richmond, CA). The LIFETEST procedure and Wilcoxon Chi-square test were used to compare survival rates of exposed and control pups. The GLM procedure was used to assess differences in weight gain in dams and pups (SAS software Version 9.4, SAS Institute Inc., Cary, NC). The 84 genes that were assessed in the array related to Th1 and Th2 responses were analyzed using the RT^2^ Profiler PCR Array data Analysis software version 3.5 (http://pcrdataanalysis.sabiosciences.com/pcr/arrayanalysis.php, SABiosciences, Qiagen). A global mean approach was used for normalization of all samples. A *p*-value less than 0.05 was considered statistically significant.

## Additional file

Additional file 1:
**Placenta histopathology.** Representative micrographs of placenta sections stained with H&E from mice exposed prenatally to Cu NPs or laboratory air (shams). No significant pathological changes were found in the placentas from exposed animals compared to the controls. Bars = 200 μm (inset, bars = 20 μm). A = labyrinth layer, B = spongiotrophoblast layer, C = decidual layer. (PDF 322 kb)
