# Supplementary figures and images for: Effects of prenatal inhalation exposure to copper nanoparticles on murine dams and offspring
Source: Part Fibre Toxicol. 2015 Oct 6;12:30. doi: 10.1186/s12989-015-0105-5 (PMC4594905; doi:10.1186/s12989-015-0105-5)

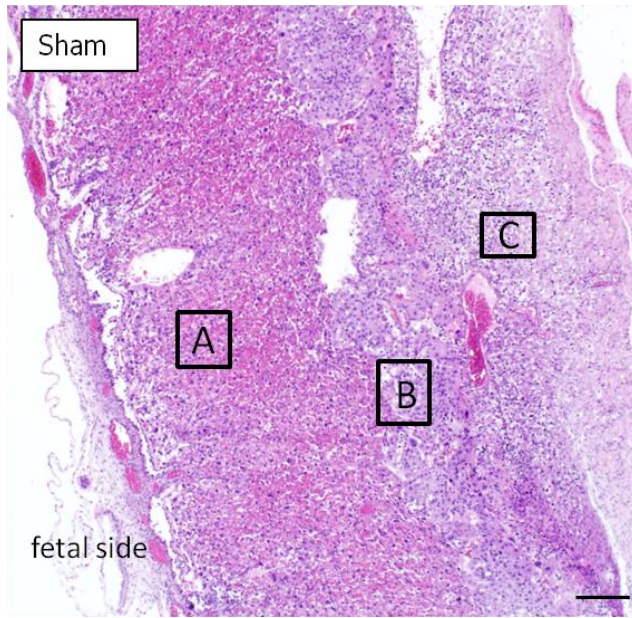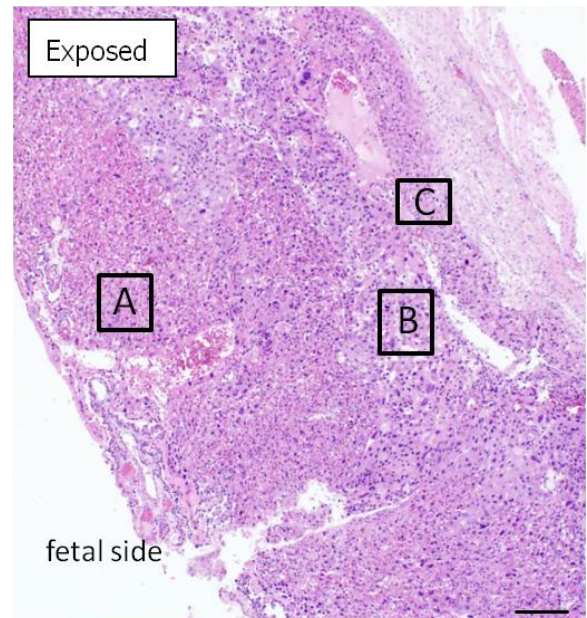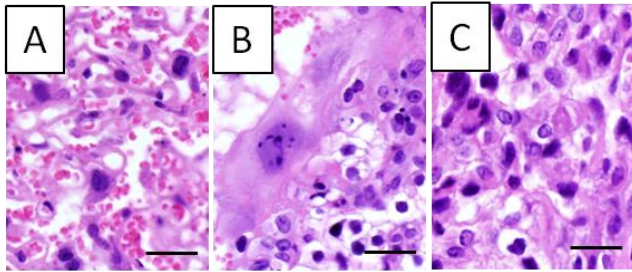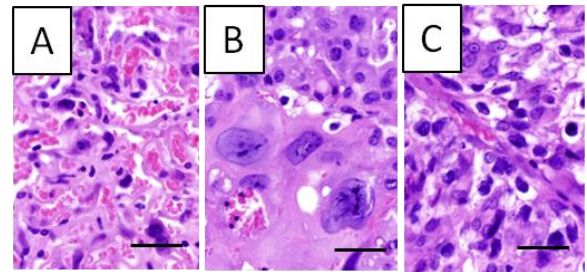

Supplement: Additional file 1: — Placenta histopathology. Representative micrographs of placenta sections stained with H&E from mice exposed prenatally to Cu NPs or laboratory air (shams). No significant pathological changes were found in the placentas from exposed animals compared to the controls. Bars = 200 μm (inset, bars = 20 μm). A = labyrinth layer, B = spongiotrophoblast layer, C = decidual layer. (PDF 322 kb) [file 12989_2015_105_MOESM1_ESM.pdf]
